# Oral Function, Loneliness, Depression, and Social Participation Among Physically Disabled Middle-Aged and Older Adult Individuals: Insights from a Japanese Cross-Sectional Study

**DOI:** 10.3390/geriatrics9050137

**Published:** 2024-10-21

**Authors:** Naoki Maki, Harumi Sakamoto, Keisuke Taniguchi, Yuhki Mutsukura, Shoko Nomura, Sechang Oh, Hisako Yanagi, Thomas Mayers

**Affiliations:** 1 Faculty of Rehabilitation, R Professional University of Rehabilitation, 2-12-31 Kawaguchi, Tsuchiura 300-0032, Japan; sakamoto@a-ru.ac.jp (H.S.); taniguchi@u.a-ru.ac.jp (K.T.); mutsukura@a-ru.ac.jp (Y.M.); nomura@a-ru.ac.jp (S.N.); o-sechan@u.a-ru.ac.jp (S.O.); yanagi@a-ru.ac.jp (H.Y.); 2 Department of Health Services Research, Institute of Medicine, University of Tsukuba, 1-1-1 Tennodai, Tsukuba 305-8575, Japan; mayers@md.tsukuba.ac.jp; 3 Medical English Communications Center, Institute of Medicine, University of Tsukuba, 1-1-1 Tennodai, Tsukuba 305-8575, Japan

**Keywords:** oral dysfunction, physically disabled, middle-aged, older adult, social engagement, social isolation, loneliness, frailty

## Abstract

**Background/Objectives**: In the context of an aging society, physical disability and its relationship with frailty is of growing concern. The aim of this study was to examine the associations between oral function, social participation, and loneliness among community-dwelling middle-aged and older adult physically disabled individuals. **Methods**: In this cross-sectional study, the participants were 140 individuals with certified physical disabilities living in the studied area. Demographic characteristics, outing activities, loneliness (Three-Item Loneliness (TIL) Scale), and frailty/ability to live independently (Kihon Checklist (KCL)) were assessed using a questionnaire survey. The participants were divided into two groups based on the presence or absence of oral dysfunction (OD), and statistical analyses were performed to compare the groups. **Results**: The group with OD had significantly higher TIL and KCL total scores and significantly lower mobility, confinement, cognitive function, greater levels of depression, and fewer outing activities (volunteering, movies, festivals, sports) compared to the group without OD. In a multivariate, age- and sex-adjusted binomial logistic regression analysis, outing activities (OR = 0.011, 95% CI: 0.000–0.529, *p* = 0.023) and loneliness (OR = 6.174, 95%CI: 1.292-29.502, *p* = 0.023) were identified as significant factors. **Conclusions**: An association was found between OD, loneliness, and social activities among middle-aged and older individuals with physical disabilities. The results suggest that future interventions should consider the relationship between oral function and factors such as depression, loneliness, social isolation, and social engagement as a means to mitigate frailty and other health and well-being concerns for physically disabled individuals.

## 1. Introduction

Approximately one billion people, or about 15% of the world’s population, live with a disability, with nearly 200 million of them experiencing significant functional difficulties [1]. Disability rates continue to be of concern, largely due to aging populations and the higher risk of disability among older adults, as well as the global incidence of chronic health conditions such as diabetes, heart disease, cancer, and mental illness. People with disabilities often face poorer health outcomes, lower educational attainment, reduced participation in economic activities, and higher poverty rates compared to those without disabilities [2,3]. One contributing factor is the barriers that people with disabilities encounter when accessing essential services such as healthcare, education, employment, transportation, and information. While these issues are particularly severe in impoverished communities or developing countries [1,4], disabled individuals living in developed countries like Japan can likewise face severe challenges.

In Japan, the 2021 White Paper on Persons with Disabilities, published by the Ministry of Health, Labour and Welfare, estimated that 4.36 million people have physical disabilities, a number that is on the rise [5]. According to the Ministry’s 2019 Comprehensive Welfare Promotion Program for Persons with Disabilities, physical activity and social interaction among individuals with physical disabilities who have underlying medical conditions are declining, raising concerns about the potential negative impact on their physical health [6].

One of the most concerning health conditions in Japan is aspiration pneumonia, which is the sixth leading cause of death in the country [7]. In frail older adult individuals, the relationship between poor swallowing function and frailty has gained significant attention [8]. Risk factors such as impaired oral swallowing function and poor nutrition increase the likelihood of aspiration, which can lead to aspiration pneumonia. Eating and swallowing dysfunctions not only elevate mortality rates but are also closely linked to psychosocial issues such as social isolation, depression, cognitive decline, and loneliness. The consequences of oral dysfunctions (OD) are far-reaching and include disruptions in recreational eating opportunities and social participation, such as conversations and communication, avoidance of socializing and going out, leading to an inactive lifestyle, decreased motivation, seclusion, depression, and cognitive decline [9,10,11].

While there is increased interest in the negative effects of OD on the health and well-being of older adults in Japan, data on the associations between OD, consequent psychosomatic disorders, and social participation among middle-aged and older adults with physical disabilities are scarce. The purpose of this study, therefore, was to explore the associations between OD and psychosocial factors, including cognitive function, depression, loneliness, and social participation, among middle-aged and older adults with physical disabilities. Given that good oral health is a key component of overall health and well-being, we hypothesized that OD would be significantly associated with adverse psychosocial outcomes, including cognitive decline, depression, loneliness, and reduced social participation, among middle-aged and older adults with physical disabilities in Japan. Such associations could have important implications for their ability to live independently within the community. The findings of this study will contribute to our understanding of the role of oral function in promoting healthy aging and quality of life in individuals with disabilities.

## 2. Materials and Methods

### 2.1. Participants

Questionnaires were mailed to 200 community-dwelling individuals aged 40 and over who were certified as physically disabled, and 140 persons (recovery rate: 70.0%) who responded were included in the study. The participants were living in a rural town located in the south of Ibaraki prefecture, Japan.

### 2.2. Data Collection

The self-administered questionnaire included demographic items of age, gender, height, weight, body mass index (BMI), and details of social participation, such as engagement in volunteer activities, local festivals, movies and entertainment, and sports activities. The questionnaire also included two instruments: the Kihon Checklist (KCL), which is used to assess the basic functioning and independence of older adults and screen for frailty [12], and the Japanese version of the Three-Item Loneliness Scale (TIL) [13]. The original TIL scale has been extensively utilized to assess loneliness in a highly streamlined format [14]. The three items (1. How often do you feel that you lack companionship? 2. How often do you feel left out? 3. How often do you feel isolated from others?) are rated as “Hardly ever” (1 point), “Some of the time” (2 points), and “Often” (3 points). Higher scores indicate greater loneliness. The Japanese version of the TIL scale has demonstrated adequate reliability and validity for assessing loneliness among Japanese populations [13].

The KCL is a validated Japanese instrument used by the Ministry of Health, Labour and Welfare to identify those at high risk of requiring long-term care [15,16]. The KCL (available in English here [17]) consists of 25 items that assess the following seven aspects: (1) activities of daily living, (2) mobility, (3) nutritional status, (4) oral function, (5) confinement, (6) cognitive function, and (7) depressive mood. Each item is scored from 0 to 1 point, with a resulting score range of 0-25 points. When interpreting the KCL scores, “Motor function impairment” was defined as a score of ≥3 from the five items related to “mobility”. “Oral function impairment” was defined as a score of ≥2 from the three items related to “oral function”. “Cognitive function decline” was defined as a score of ≥1 from the three items related to “cognitive function”. “Depressive tendency” was defined as a score of ≥2 from the five items related to “depressive mood”. “Confinement” was defined where there was a negative response to the item “Do you go out at least once a week?” A total KCL score of ≥8 was considered to indicate “frail”, meaning that the patient was at risk for loss of independence.

The participants were divided into two groups: (1) those with oral function impairment (a KCL score of ≥2 for “oral function”) were classified into the Oral Dysfunction (OD) group, and (2) the remaining participants (with a score of <2 for “oral function”) were classified into the Non-oral dysfunction (Non-OD) group.

### 2.3. Ethical Statement

The study was conducted between September 2023 and February 2024 and was approved by the Ethics Committee of R Professional University of Rehabilitation (No. R23-007).

### 2.4. Statistical Analysis

In the statistical analysis, Mann–Whitney U test, *t*-test, and chi-squared test were used to compare the two groups. Binomial logistic regression analysis (forced entry method) adjusted for age and gender was used for multivariate analysis. SPSS (IBM Statistics Ver. 26) was used for analysis, with a significance level set at 5%. The sample size calculation was based on a statistical power of 95%, an alpha level of 5%, and a medium effect size, resulting in a minimum required sample size of 89 [18]. Therefore, the final sample size of 140 in this study provides sufficient statistical power to detect a significant effect.

## 3. Results

### 3.1. Disabilities Within the Study Cohort

Table 1 shows the types of physical disabilities of the participants included in our study. “Upper and lower limb trunk and motor dysfunction” was the most common disability (*n* = 50, 35.7% participants), followed by “other” (*n* = 25, 36%) and “kidney disorder” (*n* = 10, 7.1%).

### 3.2. Participant Characteristics

Table 2 shows the participant characteristics in total and divided by the two groups. Of the 140 participants, 91 (65.0%) were female and 49 (35.0%) were male. After dividing the participants by oral dysfunction (according to KCL score), 32 (22.8%) participants were placed in the OD group (19 (54.9%) women and 13 (40.6%) men), and the remaining 108 (77.2%) participants were placed in the Non-OD group (72 (66.7%) women and 36 (33.3%) men), showing no significant difference between the two groups. The average age of participants in the OD group (75.2 ± 12.7) was significantly higher than those in the non-OD group (68.6 ± 12.9), with a *p*-value of 0.009, indicating a statistically significant difference. Older participants are more likely to have OD. A lower percentage of participants in the OD group (7.6%) engaged in outing activities compared to the non-OD group (22.0%), but this difference was not statistically significant (*p* = 0.099). Participants in the OD group had significantly higher TIL scores (4.8 ± 2.0) compared to the Non-OD group (4.3 ± 1.9), with a *p*-value of <0.001, indicating that those with OD were lonelier. The total KCL score was significantly higher in the OD group (20.4 ± 7.7) than in the Non-OD group (12.7 ± 6.7) (*p* < 0.001), reflecting poorer overall health and functioning in the OD group. Within the various components of the KCL, the OD group reported significantly worse mobility (3.2 ± 1.4) compared to the Non-OD group (1.7 ± 1.7) (*p* < 0.001), indicating greater physical limitations in participants with OD. Cognitive function scores were likewise significantly lower in the OD group (1.0 ± 1.0) compared to the Non-OD group (0.4 ± 0.7), with a *p*-value of 0.007, suggesting that participants with OD may have greater cognitive impairment. Participants in the OD group reported a higher degree of confinement (0.6 ± 0.5) compared to the Non-OD group (0.2 ± 0.5), with a *p*-value of 0.024 and significantly higher depressive mood scores (2.5 ± 1.8) than the Non-OD group (0.8 ± 1.3; *p*-value of <0.001), indicating greater levels of depression in participants with OD.

### 3.3. Logistic Regression Analysis

Table 3 shows the results of the logistic regression analysis of the factors related to OD among the study participants. The binomial logistic regression analysis with age- and sex-adjusted oral dysfunction as the dependent variable revealed a significant difference between the two groups regarding the variable “Outing activities” and “TIL Scale”. Participants who engaged in outing activities, such as volunteering, attending movies, festivals, or sports, were significantly less likely to experience oral dysfunction, with an odds ratio of 0.011 (*p* = 0.023). This indicates that participating in social and recreational activities may play a protective role in preventing oral dysfunction. Higher levels of loneliness, as measured by the TIL scale, were strongly associated with an increased likelihood of oral dysfunction, with an odds ratio of 6.174 (*p* = 0.023). This suggests that feelings of loneliness may be a significant risk factor for the development of oral dysfunction.

## 4. Discussion

The findings of this study reveal a number of concerning trends in the associations between OD, physical disability, frailty, and psychosocial factors in older adults. Firstly, our study revealed that approximately one in four participants, all of whom were certified as physically disabled, exhibited impaired oral function. Secondly, the participants without OD were significantly more frail compared to those without such impairments.

The findings of our logistic regression analysis highlight the importance of social engagement and its association with, and possible protective effect against, OD. Participants who engaged in outing activities, such as volunteering or attending social events, were significantly less likely to experience OD. This suggests a notable connection between maintaining an active lifestyle, participating in community-based activities, and oral health. While it remains unclear whether an active lifestyle directly prevents oral health decline or if individuals with better oral health tend to lead more active lives, the reciprocal nature of this relationship is evident. For example, in a survey of older adults requiring long-term care, Ishii et al. found that spending more time out of bed, going out, and experiencing a higher quality of life were linked to better eating and swallowing function [19]. They suggested that staying out of bed longer helps maintain a stable posture conducive to eating and swallowing, preserves the muscles involved in these functions, and increases the desire to eat. From their findings, they also indicated that going out and visiting the places that they like, as well as voluntarily engaging with society, may help prevent cognitive decline by alleviating chronic stress, thereby contributing to the maintenance of healthy brain function, which is also linked to better feeding and swallowing abilities [19].

Moreover, poor oral function, particularly in swallowing, has direct consequences for food intake and nutritional status, which in turn negatively affect quality of life. According to Chen et al. [20], poor oral function, particularly in swallowing, directly impacts food intake and nutritional status, which in turn negatively affects quality of life. Impaired swallowing function has been associated with poor nutritional status, physical decline, and reduced social activities such as outings and volunteer work. They further report that xerostomia and reduced tongue pressure can similarly hinder food and nutritional intake, potentially leading to a further decrease in social activities. Wu et al. [21] also found that good oral function motivates older adults to actively engage in social activities and volunteer work. They report that older adults with good oral function are more likely to maintain social interactions and tend to have better psychological health. In an Italian study of older adults, Laudisio et al. observed that masticatory dysfunction, commonly observed in older individuals, is linked to poorer nutritional status and may independently contribute to disability in older populations [22]. The findings of some studies of Japanese older adults have shown how swallowing difficulties, cognitive decline, and malnutrition had both direct and indirect impacts on reducing the ability to perform activities of daily living in older individuals receiving home nursing care [23] and how poor dental health was linked to an increased risk of developing functional disabilities [24]. Taken together, our findings, alongside those of other researchers, contribute to the evidence pointing to the importance of not only direct functional interventions to enhance eating and swallowing in community-dwelling individuals with physical disabilities but also the need for support that promotes social participation and increases social activity to improve overall quality of life.

In our study, participants in the OD group were significantly older, had worse mobility, and reported higher levels of loneliness and depressive mood compared to the Non-OD group. These factors indicate that oral dysfunction is not an isolated issue but is intricately linked to broader health outcomes, particularly in the context of aging. The significantly higher KCL scores in the OD group, reflecting poorer overall functioning and greater physical limitations, highlight the compounded impact of OD on both physical and psychosocial well-being. The greater degree of confinement and lower cognitive function observed in the OD group further suggest that OD may contribute to a vicious cycle of declining health and social withdrawal.

One of the most significant findings of our study is the strong association between loneliness and oral dysfunction. Participants in the OD group were notably lonelier, and this was reflected in the significantly higher loneliness scores observed in the multivariate analysis. This aligns with previous research linking loneliness to poor oral health, particularly in older adults [25,26,27,28], including older Japanese adult populations [29,30,31]. Loneliness has been associated with declines in oral-related QOL, such as difficulties with eating and speaking, which may further isolate individuals socially and psychologically [28,31,32,33]. In turn, the bi-directional association between social isolation and OD means that loneliness could exacerbate oral health problems, potentially creating a feedback loop that leads to further deterioration in both oral and overall health [27].

The psychosocial effects of loneliness are profound. Loneliness is not only linked to oral health deterioration but also to increased functional decline and higher mortality rates among older adults, and individuals with disabilities who experience loneliness are at even greater risk of challenges in daily living, mobility, and overall well-being [34,35,36,37]. The relationship between health and loneliness is multi-faceted and, as suggested by Hawkley and Cacioppo, it is programmed into our “evolutionary design as a social species” [38]; however, in older adults, one important association is loneliness’ link to negative health behaviors, including unhealthy practices such as increased alcohol consumption and smoking, as well as a decrease in health-promoting activities like regular physical exercise, proper nutrition, and good sleeping routine [37], all of which could contribute significantly to reduced oral health. Thus, it is important for healthcare providers to be aware of the significant impact of psychosocial factors, such as loneliness, on physical health, particularly oral health and swallowing function.

In addition, depressive mood was significantly higher in the OD group, suggesting that psychological factors are closely linked to oral health, which has been established in previous studies [31,39,40]. The association between oral dysfunction and depressive symptoms may stem from the psychological burden of living with compromised oral function, which can affect an individual’s ability to enjoy food, communicate, and maintain social relationships [41]. Depression itself may also impede oral care practices, leading to a further decline in oral health [31,39,40]. This particular topic is gaining much attention in Japan, with a number of studies investigating the relationship between oral health and depression [31,42,43,44].

Although the difference in social outings and between the OD and Non-OD groups was not statistically significant, the trend toward fewer outings in the OD group suggests that social participation plays a role in oral health. Social activities may provide protective benefits by promoting physical activity, reducing loneliness, and improving mental well-being. Encouraging older adults, especially those at risk for oral dysfunction, to participate in social activities may contribute to better oral health, improved mental health, and overall quality of life.

Research examining the differences in oral health in urban versus rural settings largely agrees that rural-dwelling older adults have worse oral health outcomes, including greater tooth loss, fewer dental visits, and poorer self-reported oral health [45,46,47]. For example, a study from Japan showed that individuals living in rural areas had less access to family dentistry than their urban-dwelling counterparts [48], which likely has implications for their oral functioning. Additionally, a study from China exploring health and quality of life outcomes in urban- versus rural-dwelling individuals with disabilities shows inequalities, including poor self-reported health, functional disabilities, and depression [49]. Addressing disparities in access to dentistry and education around good oral health practice for rural-dwelling older adults, especially those with disabilities, could help to maintain their overall quality of life. Interestingly, some evidence suggests that there is a higher prevalence of perceived social isolation [50] and depression [51] amongst urban-dwelling older adults. Therefore, it is important for healthcare providers to be aware of the different challenges faced by older adults living with disabilities in rural and urban contexts.

Overall, our study demonstrates that oral dysfunction is closely linked to frailty, loneliness, and cognitive decline in physically disabled older adults. These findings call for integrated healthcare approaches that address both oral health and broader physical, mental, and social well-being. It is important to state that oral health is not the sole purview of dentistry but rather should form a crucial part of general health assessments and healthcare interventions, and recent studies are emphasizing the need for a multidisciplinary approach to oral health [52,53,54]. For example, in a previous randomized control trial, we found that the inclusion of respiratory rehabilitation exercises alongside regular rehabilitation in frail older patients not only significantly improved respiratory function but also swallowing function and quality of life compared to controls [55], demonstrating that physical therapists can play a role in the oral health of at-risk individuals. Thus, healthy aging strategies should focus holistically on improving oral care, promoting physical activity, enhancing social engagement, and providing mental health support, particularly for individuals at risk of oral dysfunction due to age or physical disability. Given the aging nature of the population, early intervention is crucial, and efforts should perhaps begin in younger demographics to prevent frailty and oral health deterioration later in life [56].

### Limitations

This study has several limitations. Although the subjects had physical disabilities and underlying medical conditions, it was challenging to examine detailed data such as the duration of the disease, the nature and course of treatment, and the extent of the condition. The small sample size limited the ability to explore the effects of stratification by age, gender, and other confounding factors in detail, and the fact that the study was conducted in a single region makes it difficult to generalize the findings. Furthermore, it should be noted that over 75% of the participants in this study were in a frail condition, which introduces a potential bias due to the population imbalance. While our data have been gathered using validated instruments, our study is limited by the fact that it relies on self-reported measures without any physical performance testing or more objective observations, and the cross-sectional design of the study limits our ability to address issues of causality. Lastly, it was challenging to assess the involvement of caregivers, as well as the frequency and specifics of outings, such as volunteer activities, movies, festivals, and sports. Further research is needed, incorporating more objective testing and measures, to explore the causal pathways between oral health and these other factors, as well as to assess effective interventions that can improve overall health and well-being in aging populations.

## 5. Conclusions

In this study, we identified an association between oral dysfunction, loneliness, depression, and participation in social activities among middle-aged and older individuals with disabilities living in a rural community. These factors may hinder the ability of physically disabled individuals to live independently and might contribute to the hastening of frailty. Healthcare providers should be cognizant of the relationship between oral function and psychological aspects such as depression, loneliness, social isolation, and participation in outgoing activities, particularly among disabled or older individuals. Addressing these elements could be crucial for mitigating the risk of aspiration pneumonia and frailty and a range of other health and quality of life-related issues among physically disabled persons in community settings.

## Figures and Tables

**Table 1:** Type of physical disabilities.

	Participants (*n* = 140) *n* (%)
Visual impairment	9 (6.4%)
Hearing impairment	9 (6.4%)
Balance dysfunction	5 (3.5%)
Speech and mastication dysfunction	9 (6.4%)
Upper and lower limb trunk and motor dysfunction	50 (35.7%)
Heart disorder	7 (5.0%)
Kidney disorder	10 (7.1%)
Liver disorder	2 (1.4%)
Respiratory disorder	7 (5.0%)
Bladder disorder	0 (0.0%)
Rectal disorder	5 (3.5%)
Small intestine disorder	1 (0.7%)
Other	36 (25.7%)

**Table 2:** Participant characteristics (total and by group).

Characteristics	Total	Non-OD Group	OD Group	Effect Size	*p*
	(*n* = 140)	(*n* = 108)	(*n* = 32)		
Age (41–93 years)	70.1 ± 4.3	68.6 ± 12.9	75.2 ± 12.7	0.34	0.009 **
Sex: Female (%)	91 (65.0%)	72 (66.7%)	19 (54.9%)	0.19	0.448
Male (%)	49 (35.0%)	36 (33.3%)	13 (45.1%)		
BMI (kg/m^2^)	22.1 ± 3.8	22.9 ± 2.5	21.2 ± 3.1	0.21	0.357
Outing activities				0.26	0.099
Yes (%)	21 (18.7%)	19 (22.0%)	2 (7.6%)		
No (%)	91 (81.3%)	67 (78.0%)	24 (92.3%)		
TIL Scale	4.5 ± 1.9	4.3 ± 1.9	4.8 ± 2.0	0.43	<0.001 **
KCL (total score)	14.8 ± 7.8	12.7 ± 6.7	20.4 ± 7.7	0.43	<0.001 **
Mobility	2.1 ± 1.8	1.7 ± 1.7	3.2 ± 1.4	0.43	<0.001 **
Nutritional status	0.2 ± 0.4	0.1 ± 0.3	0.2 ± 0.4	0.23	0.241
Oral function	0.7 ± 0.9	0.3 ± 0.4	2.3 ± 0.4	0.43	<0.001 **
Confinement	0.5 ± 0.7	0.2 ± 0.5	0.6 ± 0.5	0.31	0.024 *
Cognitive function	0.6 ± 0.8	0.4 ± 0.7	1.0 ± 1.0	0.34	0.007 **
Depressive mood	1.2 ± 1.6	0.8 ± 1.3	2.5 ± 1.8	0.43	<0.001 **
Frail				0.43	<0.001 **
Yes (%)	86 (75.4%)	56 (67.5%)	30 (96.8%)		
No (%)	28 (24.6%)	27 (32.5%)	1 (3.2%)		

* *p* < 0.05; ** *p* < 0.01; Mean ± SD; *n* (%); Chi-square test; Mann–Whitney U test; *t*-test; OD: Oral dysfunction; BMI: body mass index TIL: Japanese version of the Three-Item Loneliness Scale; KCL: Kihon Checklist.

**Table 3:** Logistic regression analysis of factors related to oral dysfunction.

Independent Variables	Odds Ratio	95%CI (LL–UL)	Effect Size	*p*
Outing activities (volunteer, movies, festivals, sports)	0.011	0.000–0.529	<0.01	0.023 *
UCLA-J	6.174	1.292–29.502	0.83	0.023 *
Mobility	0.059	0.000–9.667	<0.01	0.277
Confinement	0.005	0.000–2.605	<0.01	0.097
Cognitive function	45.589	0.229–9067.236	2.12	0.157
Depressive mood	104.654	0.472–23,190.799	6.23	0.091

* *p* < 0.05; Binomial logistic regression analysis adjusted for age and gender was used for multivariate analysis. CI: confidence interval; LL: Lower Limit; UL: Upper Limit; TIL: Japanese version of the Three-Item Loneliness Scale.

## Data Availability

The data presented in this study are available upon request from the corresponding author due to issues of privacy.
